# Gaining traction: Promising shifts in gender norms and intimate partner violence in the context of a community-based HIV prevention trial in South Africa

**DOI:** 10.1371/journal.pone.0237084

**Published:** 2020-08-20

**Authors:** Ann Gottert, Julie Pulerwitz, Nicole Haberland, Rhandzekile Mathebula, Dumisani Rebombo, Kathryn Spielman, Rebecca West, Aimée Julien, Rhian Twine, Dean Peacock, Mi-Suk Kang Dufour, F. Xavier Gómez-Olivé, Audrey Pettifor, Sheri A. Lippman, Kathleen Kahn

**Affiliations:** 1 Population Council/Project SOAR, Washington, D.C., United States of America; 2 Sonke Gender Justice, Bushbuckridge Local Municipality, South Africa; 3 Independent Consultant, Johannesburg, South Africa; 4 Division of Prevention Science, Department of Medicine, University of California San Francisco, San Francisco, CA, United States of America; 5 Department of Epideiology, University of North Carolina at Chapel Hill, Chapel Hill, NC, United States of America; 6 MRC/Wits Rural Public Health and Health Transitions Research Unit (Agincourt), School of Public Health, Faculty of Health Sciences, University of the Witwatersrand, Johannesburg, South Africa; 7 Promundo, Washington, D.C., United States of America; 8 University of Cape Town School of Public Health, Cape Town, South Africa; University of Cape Town, SOUTH AFRICA

## Abstract

**Background:**

HIV and violence prevention programs increasingly seek to transform gender norms among participants, yet how to do so at the community level, and subsequent pathways to behavior change, remain poorly understood. We assessed shifts in endorsement of equitable gender norms, and intimate partner violence (IPV), during the three-year community-based trial of *Tsima*, an HIV ‘treatment as prevention’ intervention in rural South Africa.

**Methods:**

Cross-sectional household surveys were conducted with men and women ages 18–49 years, in 8 intervention and 7 control communities, at 2014-baseline (n = 1,149) and 2018-endline (n = 1,189). Endorsement of equitable gender norms was measured by the GEM Scale. Intent-to-treat analyses assessed intervention effects and change over time. Qualitative research with 59 community members and 38 staff examined the change process.

**Results:**

Nearly two-thirds of men and half of women in intervention communities had heard of the intervention/seen the logo; half of these had attended a two-day workshop. Regression analyses showed a 15% improvement in GEM Scale score over time, irrespective of the intervention, among men (p<0.001) and women (p<0.001). Younger women (ages 18–29) had a decreased odds of reporting IPV in intervention vs. control communities (aOR 0.53; p<0.05). Qualitative data suggest that gender norms shifts may be linked to increased media access (via satellite TV/smartphones) and consequent exposure to serial dramas modeling equitable relationships and negatively portraying violence. Tsima’s couple communication/conflict resolution skills-building activities, eagerly received by intervention participants, appear to have further supported IPV reductions.

**Conclusions:**

There was a population-level shift towards greater endorsement of equitable gender norms between 2014–2018, potentially linked with rapid escalation in media access. There was also an intervention effect on reported IPV among young women, likely owing to improved couple communication. Societal-level gender norm shifts may create enabling environments for interventions to find new traction for violence and HIV-related behavior change.

## Introduction

Propelled by calls that gender inequities must be addressed for HIV and intimate partner violence (IPV) prevention efforts to be successful [1–3], interventions seeking to transform gender norms in order to prevent IPV and HIV are on the rise. Such interventions often revolve around participatory group-based activities, and sometimes integrate community mobilization and/or mass media approaches. Many have now been evaluated via rigorous study designs including randomized controlled trials [4–10]. Systematic reviews of these evaluations have concluded that the interventions hold promise in shifting gender norms and, in many cases behaviors, among men and women in the areas of IPV and HIV prevention as well as reproductive and child health [11–13]. Recently, interventions are also seeking to shift gender attitudes and norms as one pathway to reach the UNAIDS goals for HIV testing, treatment and viral suppression [14–18].

Yet comparably little is known about how to effect meaningful changes in gender norms–social expectations about men’s and women’s appropriate roles, rights, and responsibilities–at the community and societal levels, outside of group-based activities. According to Barker et al.’s 2010 review of gender-transformative interventions, “More evidence is needed…on how to achieve large scale and sustained reach necessary to change gender norms and power dynamics…” [13, p. 549]. Similarly, Jewkes et al. (2015) described a necessary conceptual shift towards “approaches that seek to transform the relations, social norms, and systems that sustain gender inequality and violence” [19, p. 1580]. While interventions have consistently produced notable changes among direct participants, their effects on community-level norms are often weaker or remain unclear [8,11]. And, when community-level changes in gender norms do occur, a clearer understanding is needed of how and under what circumstances this translates into changes in IPV and HIV-related behaviors among men and women. For example, recent trials of community-based gender transformative interventions in South Africa have shown mixed results regarding effects on decreasing IPV, even if effects on reducing harmful gender norms were demonstrated [7,20,21]. Finally, important questions remain about the sustainability of shifts in community-level norms and behavior, after intervention activities end [10,11,13,22]. By and large there have been few funding or policy commitments to implement such programming at scale over the long-term, with the encouraging recent exceptions of their integration within large-scale initiatives to reduce HIV vulnerability among adolescent girls and young women [23–25].

Perhaps nowhere is the need to answer these questions more evident than in communities heavily impacted by twin HIV-IPV epidemics, such as in South Africa [1–3]. In this paper, we investigate whether and how gender norms and IPV changed during a three-year cluster randomized controlled trial (RCT) of an HIV ‘treatment as prevention’ community mobilization intervention to address social barriers to HIV service use in Mpumalanga province, South Africa, where nearly one-quarter of the population is living with HIV [26]. We draw on pre-post cross-sectional surveys implemented in 2014 and 2018 with large population-representative, household-based samples of men and women, complemented by qualitative research with community members and intervention staff.

## Materials and methods

### Study setting

This study was conducted in the Agincourt Health and Socio-Demographic Surveillance System (Agincourt HDSS) area run by the Medical Research Council/Wits University Rural Public Health and Health Transitions Research Unit (Agincourt), where an annual household census has been conducted since 1992 [27]. The Agincourt HDSS is located in the Bushbuckridge sub-district in Mpumalanga, a largely rural province characterized by high levels of poverty, unemployment, labor migration, and HIV infection [27,28].

### Parent study trial and intervention

The study described in this paper was part of a community RCT to test the effects of ‘Community Mobilization for Treatment as Prevention’, a three-year intervention to increase HIV testing, linkage to and retention in care and treatment among men and women ages 18–49, by addressing key social barriers to service uptake–including inequitable gender attitudes and norms. For the trial, eight communities were randomized to receive the intervention, and seven to control/no intervention [15]. Results regarding intervention effects on the primary trial endpoints (i.e., HIV testing, linkage to and retention in care) are forthcoming.

The community mobilization intervention, *Tsima* (“working together”), was based on a defined mobilization model [29] and has been described in detail elsewhere [15]. Tsima was implemented by Sonke Gender Justice, a South African NGO. It was adapted from Sonke’s program *One Man Can*, with substantial changes made in order to (1) add more emphasis on HIV testing and treatment uptake (the main trial endpoints), and (2) equally engage and engender change among both men (the main target population for *One Man Can*) and women. Intervention staff included two program managers, a team of 18 Community Mobilizers with extensive training from master trainers at Sonke, and volunteer Community Action Team (CAT) members. Intervention content covered six themes: *Gender*, *power and health; HIV prevention*: *Community knowledge is community power; Treatment literacy; Healthy communication*, *healthy relationships; Human rights*, *HIV and stigma; and Taking action for change*. Activities comprised two-day workshops (with about 10–30 male and female participants), community-based activities (e.g., street theatre; murals; meetings with local leaders), young women’s groups, support groups for people living with HIV (PLHIV), and engaging village leadership and other stakeholders. Each of the five two-day workshop curricula (of which most participants completed multiple), and many of the other activities, intentionally integrated critical reflection and testimonials about inequitable gender norms and relationship violence, including links with HIV risk and engaging in testing, care and treatment services. Several workshop sessions also aimed to build couple communication and conflict resolution skills (e.g., learning and role-playing healthier communication skills like assertive communication, talking more openly with partners about safer sex and HIV, and supporting HIV status disclosure).

### Survey sample and procedures

Quantitative data come from two cross-sectional surveys: a baseline survey conducted in control and intervention communities pre-intervention from July-November 2014, and an endline survey conducted from July-December in 2018. The sampling frame consisted of all HDSS households with a resident aged 18–49 enumerated during the Agincourt HDSS from 2013 (for baseline) or 2017 (for endline). In order to generate adequate sampling frames for each sex in each community, each household was designated for either a male or female participant based on HDSS data. Only one person was interviewed per household.

The survey was administered in the participant’s home by a trained interviewer, who read each question to the respondent then entered his/her answer into an electronic form on a tablet computer. The survey was conducted in the local language of Shangaan or in English, and lasted one to two hours.

#### Measures

**Endorsement of equitable gender norms** was measured by the Gender Equitable Men (GEM) Scale [30], which has been previously employed in the study site [7,31]. Response categories for each of the 23 items in the scale were “Agree a lot”, “Somewhat agree,” and “Do not agree at all.”

A ‘composite’ variable was created that included all items (23 items). Variables for four sub-dimensions conceptualized as being critical to uptake of HIV testing and treatment [18] were also constructed: norms condoning men’s violence and control over women (7 items; e.g., “A man is expected to discipline his woman”); norms around men as decision-maker in a couple (6 items, e.g., “A man should have the final word about decisions in his home”); norms around men’s toughness and avoidance of help-seeking (5 items, e.g., “For men, getting sick is a sign of weakness”); and norms around women’s primary responsibility as family caretaker (5 items, e.g., “A woman’s role is taking care of her home and family”).

The scale was scored by taking the mean of non-missing items, and re-scaled for interpretability, with scores ranging from 0 to 10 and higher scores representing endorsement of more equitable gender norms. The composite scale as well as each sub-dimension had good internal reliability, with Ordinal Theta (similar to Cronbach’s alpha [32]) of 0.88 for the composite score and ≥0.70 for each sub-dimension [18].

**IPV perpetration (among male respondents) or experience (among female respondents)** was defined as reporting perpetrating or experiencing at least one of seven types of physical or sexual IPV in the last year, on a World Health Organization questionnaire adapted for South Africa [33,34]. Only individuals who had an intimate partner were asked these questions (83% of men and 89% of women); those who did not were coded as missing.

#### Statistical analysis

All data analyses were performed separately for men and women using Stata v15 [35]. Linear regression models were constructed for GEM Scale score (composite and each sub-dimension), and logistic regression models for IPV. These analyses followed an intent-to-treat (ITT) approach and were conducted both on the full age range, and for ages 18–29 and 30–49 separately. Each model included intervention status (intervention or control), timepoint (baseline or endline), and the interaction between intervention and timepoint. The resulting coefficients included the effect estimate for timepoint (i.e., change over time regardless of intervention status) and the coefficient for intervention status by time, which reflects the additional difference in changes over time by study arm (i.e., intervention effect) [7,15]. Robust standard errors were used (via the SVY command in Stata) to account for any clustering by community [35]. Models were adjusted for demographic variables hypothesized to be associated with outcomes: age, education, marital status and earning income in the last year.

All analyses of data from this population-representative survey were weighted (using scaled weights) to account for sampling probability and to represent the distribution of men and women aged 18–49 years based on Agincourt HDSS censuses conducted in 2013 (for 2014 baseline) and 2017 (for 2018 endline). Weighting procedures followed the parent study sampling weights protocol and are similar to procedures adopted in other recent community RCTs [36]. In the results section we report the few notable differences in findings between weighted and unweighted analyses.

### Qualitative methods

In-depth interviews (IDIs) were conducted with 23 intervention staff (community mobilizers and CAT members) and 59 community members, as well as two focus group discussions (FGDs) with a total of 11 community mobilizers (10 of whom completed previous IDIs). Qualitative research included in this analysis was conducted at two time-points: in Year 3 of the three-year intervention (in 2018), and in 2019, after preliminary endline survey data analyses were complete. Semi-structured guides were used in all interviews/discussions. IDI guides used in Year 3 of the intervention included questions about changes in gender norms and IPV among men and women over the last three years, including effects of the intervention. Post-endline IDI and FGD guides covered possible reasons for the survey evaluation findings related to changes in gender norms and IPV, based on reviews of the literature and discussions with local stakeholders. These questions were phrased in an unbiased/non-leading manner first (in order to minimize bias in results), then followed by more directed questions.

All IDIs and FGDs were held in a private location, conducted in English or Shangaan, and audio-recorded then transcribed and translated from Shangaan into English. Coding of transcripts was performed in atlas.ti v7 software by two coders (with six transcripts double-coded and coding differences discussed until consensus was reached). Code reports were then reviewed, with constant comparison between responses of community members, intervention staff, and other key informants (e.g., community leaders), and thematic analysis was used as a framework to arrive at final themes [37].

### Ethics

All study participants provided written informed consent to participate. The study was approved by Institutional Review Boards at the University of California-San Francisco, the University of North Carolina at Chapel Hill, the Human Research Ethics Committee at the University of the Witwatersrand in South Africa, and the Mpumalanga Department of Health and Social Development Research Committee.

## Results

### Sample characteristics

A total of 1,149 individuals in the 15 study communities completed surveys at baseline (566 men; 583 women), and 1,189 individuals at endline (541 men; 648 women). Twenty-four individuals (2%) at baseline and 89 (7%) at endline refused participation in the survey, primarily due to lack of time. Descriptive and inferential statistics presented below are weighted. Unweighted findings (available upon request) were similar; the few statistically significant differences are noted below.

Intervention and control communities were similar with respect to key socio‐demographic characteristics at baseline and, to a lesser degree, at endline (Table 1). These socio-demographic characteristics were controlled for in all regression analyses. There were no statistically significant differences in gender norms or IPV at baseline (shown in Tables 2 and 4, respectively), except that men in intervention communities endorsed slightly more equitable gender norms than those in control communities (5.79 vs. 5.42; p<0.05).

**Table 1:** Respondent characteristics by sex at baseline and endline, stratified by study arm.

	Baseline (2014)			Endline (2018)			
	N = 1,149			N = 1,189			
	Control	Intervention		Control	Intervention		p-value for diff. by timepoint (irrespective of intervention)
	n = 539	n = 610		n = 558	n = 631		
	Weighted %	Weighted %	p-value	Weighted %	Weighted %	p-value	
	(95% CI)	(95% CI)		(95% CI)	(95% CI)		
**MEN**	n = 265	n = 301		n = 258	n = 283	
Age (mean)	29.6	28.6	ns	29.6	29.3	ns	ns
	(28.0, 31.2)	(27.9, 29.4)		(28.6, 30.5)	(28.2, 30.4)		
Completed high school	30.5	35.9	ns	48.0	35.3	***	ns
	(24.4, 37.3)	(26.3, 46.8)		(44.5, 51.5)	(30.7, 40.3)		
Married (vs. other)	20.3	26.3	ns	16.9	24.0	*	ns
	(15.5, 26.0)	(22.4, 30.7)		(13.1, 21.5)	(19.5, 29.2)		
Received any income in past three months	28.0	36.3	ns	30.6	37.5	ns	ns
	(23.0, 33.5)	(27.1, 46.7)		(27.8, 33.7)	(30.1, 45.6)		
**WOMEN**	n = 274	n = 309		n = 300	n = 348		
Age (mean)	32.8	32.7	ns	33.2	33.1	ns	ns
	(32.0, 33.5)	(31.7, 33.6)		(32.8, 33.5)	(32.6, 33.6)		
Completed high school	30.3	27.1	ns	47.8	43.8	ns	***
	(25.0, 36.2)	(19.7, 36.0)		(42.2, 53.6)	(33.0, 55.2)		
Married (vs. other)	41.2	41.9	ns	39.0	34.8	ns	ns
	(35.7, 47.0)	(37.2, 46.8)		(34.8, 43.4)	(25.8, 45.0)		
Received any income in past three months	33.4	40.0	ns	54.4	44.5	**	*
	(26.7, 40.9)	(34.9, 45.4)		(51.6, 57.1)	(38.4, 50.8)		

*p<0.05

**p<0.01

*** p<0.001, ns = non-signifiant.

**Table 2:** Endorsement of equitable gender norms at baseline and endline, stratified by study arm, and adjusted effect estimates.

*GEM Scale Score*, *composite (range 0–10; higher = more equitable)*	Weighted means (95% CI)							
	BASELINE (2014)		ENDLINE (2018)		Unadjusted Beta		Adjusted Beta^a^	
	N = 1,149		N = 1,174^b^					
**MEN**	**Control**	**Interv.**	**Control**	**Interv.**	Effect of time	Effect of time x Intervention	Effect of time	Effect of time x Intervention
	n = 265	n = 301	n = 258	n = 278				
**Overall**	5.42	5.79	7.19	7.06	1.78***	-0.51	1.66***	-0.42
	(5.15, 5.68)	(5.58, 6.00)	(6.77, 7.62)	(6.51, 7.60)	(1.38, 2.18)	(-1.14, 0.12)	(1.27, 2.05)	(-1.03, 0.18)
	n = 144	n = 177	n = 163	n = 173				
**Younger (ages 18–29 years)**	5.48	5.77	6.92	7.11	1.44***	-0.10	1.33***	0.01
	(5.18, 5.79)	(5.54, 6.00)	(6.36, 7.48)	(6.40, 7.81)	(0.78, 2.09)	(-0.97, 0.77)	(0.65, 2.01)	(-0.87, 0.88)
	n = 121	n = 124	n = 95	n = 105				
**Older (ages 30–49 years)**	5.33	5.82	7.57	7.00	2.24***	-1.07**	2.08***	-0.95**
	(4.93, 5.73)	(5.50, 6.15)	(6.88, 8.26)	(6.65, 7.35)	(1.66, 2.82)	(-1.82, -0.32)	(1.60, 2.56)	(-1.60, -0.30)
**WOMEN**	**Control**	**Interv.**	**Control**	**Interv.**	Effect of time	Effect of time x Intervention	Effect of time	Effect of time x Intervention
	n = 274	n = 309	n = 300	n = 338				
**Overall**	5.96	6.05	7.42	7.55	1.46***	0.05	1.25***	0.11
	(5.71, 6.22)	(5.81, 6.28)	(7.04, 7.80)	(7.27, 7.84)	(1.02, 1.90)	(-0.43, 0.53)	(0.76, 1.74)	(-0.40, 0.61)
	n = 126	n = 136	n = 117	n = 149				
**Younger (ages 18–29 years)**	6.04	6.34	7.30	7.44	1.25***	-0.15	0.93**	0.02
	(5.89, 6.20)	(5.82, 6.86)	(6.77, 7.83)	(6.89, 7.98)	(0.76, 1.75)	(-0.76, 0.45)	(0.37, 1.50)	(-0.61, 0.66)
	n = 148	n = 173	n = 183	n = 189				
**Older (ages 30–49 years)**	5.90	5.83	7.49	7.63	1.58***	0.22	1.38***	0.26
	(5.53, 6.28)	(5.60, 6.06)	(7.12, 7.85)	(7.43, 7.83)	(1.03, 2.13)	(-0.38, 0.81)	(0.81, 1.96)	(-0.37, 0.88)

* p<0.05

** p<0.01

*** p<0.001 Values in parentheses are 95% confidence intervals.

^a^Controlling for age, education, marital status and received any income in the last three months, and adjusting for the survey sampling design.

^b^At endline, 15 respondents were missing values on all GEM Scale items, due to a survey administration error, resulting in a final sample size of 1,174.

“Effect of time” indicates change over time irrespective of the intervention. “Effect of time x Intervention” is the intervention effect—the difference in change over time in intervention vs. control communities. Effect of intervention vs. control irrespective of time is not shown, as this coefficient is not very meaningful and there were few significant findings; these findings are available from the investigators upon request.

Turning to the qualitative sample, the 23 intervention staff (i.e., mobilizers and CAT members) interviewed were 30 years old on average (range 22–44), and 26% male. The 59 community members interviewed were 36 years old on average (range 20–53), and 66.1% male. Of the 11 mobilizers who participated in the two FGDs, five were men, and they were 31 years old on average (range 25–40).

### Intervention exposure

Robust participation in Tsima was reported at endline among both men and women. In intervention communities, 64.1% of men and 56.3% of women had ever heard of Tsima or seen the Tsima logo. Of these, nearly half of men (48.1%) and women (42.0%) had participated in at least one Tsima 2-day workshop, and nearly half of men (46.4%) and over one-third of women (37.9%) reported feeling part of Tsima. Finally–also among those who had ever heard of Tsima or seen the logo–nearly two-thirds of men and women (62.8% and 66.4%, respectively) reported that due to Tsima, they had spoken with others about a Tsima-related topic (similar to those listed under themes above); around half specifically about issues around gender or relationships between men and women.

Exposure to the intervention was low in control communities. About 15% of men and women had ever heard of Tsima or seen the logo, and among them direct participation was minimal–for example, fewer than five men and five women in control communities reported attending a Tsima workshop.

### Gender norms

At baseline, GEM Scale scores fell in the middle of the range and were similar between men and women. Results of weighted bivariate and multivariable regression analyses for the GEM Scale composite score are presented in Table 2; results for the GEM Scale sub-dimensions are included in Table 3. Because bivariate results were similar, we describe multivariable regression results below.

**Table 3:** Endorsement of equitable gender norms subdimensions at baseline and endline, stratified by study arm, and adjusted effect estimates.

	Weighted means (95% CI)							
	BASELINE (2014)		ENDLINE (2018)		Unadjusted Beta		Adjusted Beta^a^	
	N = 1,149		N = 1,174^b^					
*GEM Scale Score sub-dimensions (range 0–10; higher = more equitable)*	Control	Interv.	Control	Interv.	Effect of time	Effect of time x Intervention	Effect of time	Effect of time x Intervention
**MEN**	n = 265	n = 301	n = 258	n = 278				
Norms condoning men’s violence and control over women	5.56	5.66	7.17	6.96	1.62***	-0.31	1.53***	-0.25
	(5.26, 5.86)	(5.48, 5.84)	(6.60, 7.74)	(6.22, 7.71)	(1.10, 2.13)	(-1.21, 0.59)	(1.02, 2.03)	(-1.13, 0.63)
Norms discouraging couple communication and joint decision-making	5.30	5.95	7.36	7.33	2.05***	-0.68	1.87***	-0.56
	(4.99, 5.61)	(5.73, 6.17)	(6.91, 7.80)	(6.88, 7.77)	(1.46, 2.65)	(-1.43, 0.07)	(1.27, 2.47)	(-1.26, 0.8)
Norms around men’s toughness and avoidance of help-seeking	7.13	7.67	8.50	8.41	1.37***	-0.63	1.30***	-0.56
	(6.83, 7.43)	(7.22, 8.12)	(8.12, 8.87)	(8.06, 8.75)	(0.97, 1.77)	(-1.48, 0.22)	(0.94, 1.66)	(-1.36, 0.24)
Norms around women’s primary responsibility as taking care of family	3.65	3.91	5.72	5.52	2.08***	-0.47	1.95***	-0.37
	(3.25, 4.04)	(3.20, 4.63)	(5.32, 6.12)	(4.85, 6.19)	(1.68, 2.47)	(-0.96, 0.03)	(1.56, 2.33)	(-0.84, 0.11)
**WOMEN**	n = 274	n = 309	n = 300	n = 338				
Norms condoning men’s violence and control over women	6.24	6.19	7.68	7.94	1.44***	0.31	1.20***	0.38
	(5.85, 6.64)	(5.96, 6.41)	(7.26, 8.11)	(7.74, 8.14)	(0.83, 2.05)	(-0.31, 0.93)	(0.57, 1.83)	(-0.25, 1.00)
Norms discouraging couple communication and joint decision-making	5.95	5.92	7.72	7.75	1.77***	0.06	1.50***	0.13
	(5.69, 6.21)	(5.70, 6.14)	(7.39, 8.05)	(7.04, 8.09)	(1.39, 2.15)	(-0.41, 0.52)	(1.04, 1.96)	(-0.45, 0.72)
Norms around men’s toughness and avoidance of help-seeking	7.69	7.71	8.51	8.69	0.82**	0.16	0.65*	0.19
	(7.42, 7.97)	(7.47, 7.94)	(8.06, 8.96)	(8.40, 8.98)	(0.30, 1.33)	(-0.46, 0.79)	(0.06, 1.24)	(-0.49, 0.87)
Norms around women’s primary responsibility as taking care of family	3.84	4.33	5.60	5.65	1.76***	-0.44	1.63***	-0.40
	(3.55, 4.13)	(3.68, 4.98)	(5.26, 5.95)	(5.24, 6.06)	(1.32, 2.20)	(-1.01, 0.13)	(1.14, 2.12)	(-0.94, 0.14)

* p<0.05

** p<0.01

*** p<0.001 Values in parentheses are 95% confidence intervals.

^a^Controlling for age, education, marital status and received any income in the last three months, and adjusting for the survey sampling design.

^b^At endline, 15 respondents were missing values on all GEM Scale items, due to a survey administration error, resulting in a final sample size of 1,174.

“Effect of time” indicates change over time irrespective of the intervention. “Effect of time x Intervention” is the intervention effect—the difference in change over time in intervention vs. control communities.

Among men, there was a positive association between timepoint and GEM Scale score overall (adj. Beta: 1.66; 95% CI: 1.27, 2.05; p<0.001) (Table 2). This equates to a roughly 17% increase in endorsement of equitable gender norms between baseline and endline, which was experienced similarly across intervention and control communities. Older men (ages 30–49) in intervention communities experienced less of an increase over time than those in control communities, leading to a negative intervention effect (adj. Beta: -0.95; 95% CI: -1.60, -0.30; p<0.01) (although this was non-significant in unweighted analyses; adj. Beta: -0.63, 95% CI: -1.34, 0.07). Increases over time (irrespective of intervention) also occurred for each of the four GEM Scale sub-dimensions; each with magnitudes over 10% (Table 3).

Among women, in multivariable analyses, similar to men, there was a positive association between timepoint and GEM Scale score overall (adj. Beta: 1.25; 95% CI: 0.76, 1.74; p<0.001) (Table 2). This equates to about a 13% increase in endorsement of equitable gender norms between baseline and endline, which again was experienced similarly across intervention and control communities. This increase was significantly larger for older than younger women (p value for difference: <0.05). There was also an association between timepoint and all four GEM Scale score sub-dimensions, with over a 10% increase in three of them (Table 3).

**Fig 1:**
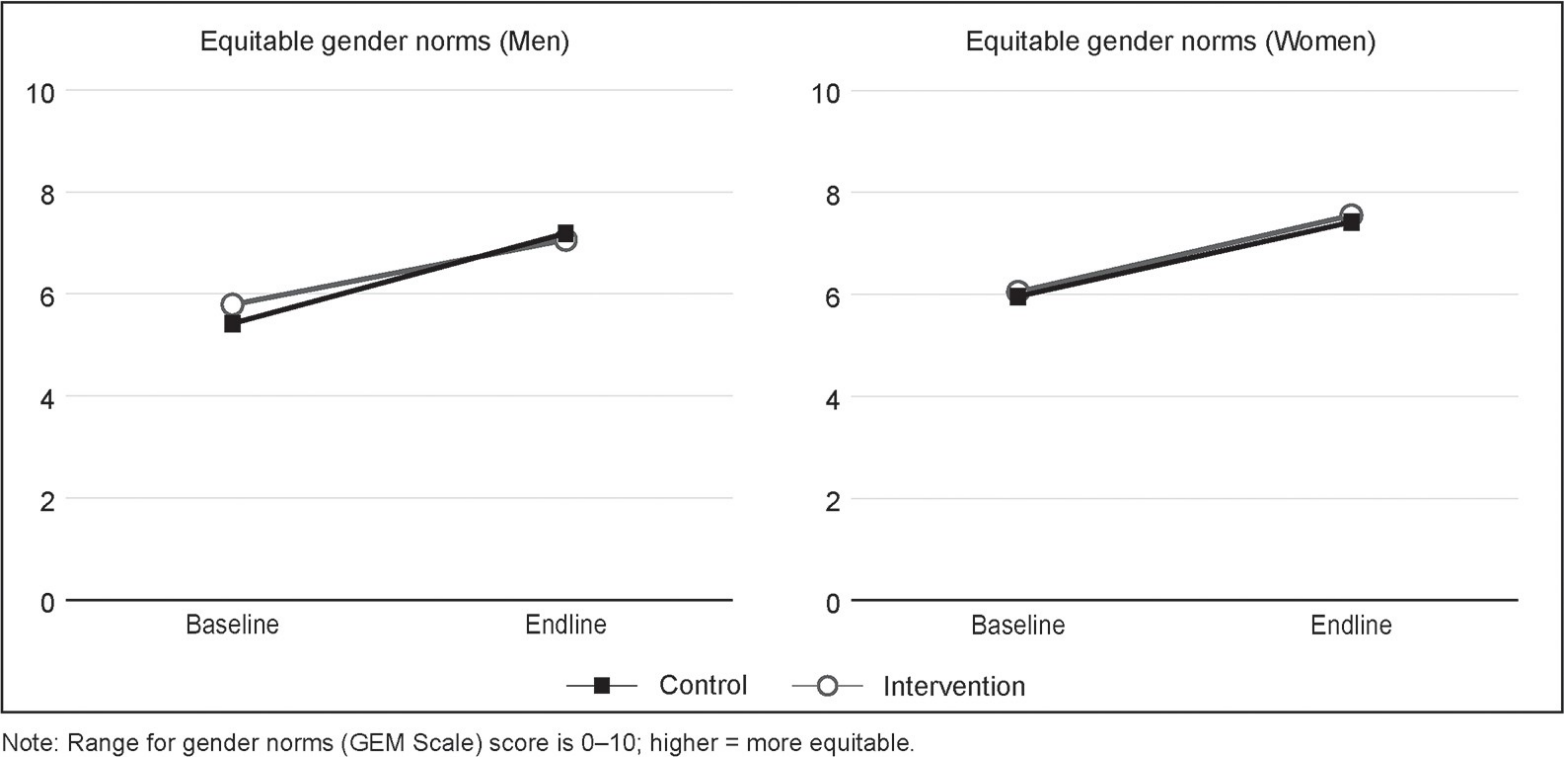
Change over time in endorsement of equitable gender norms in intervention and control communities, among men and women.

**Fig 2:**
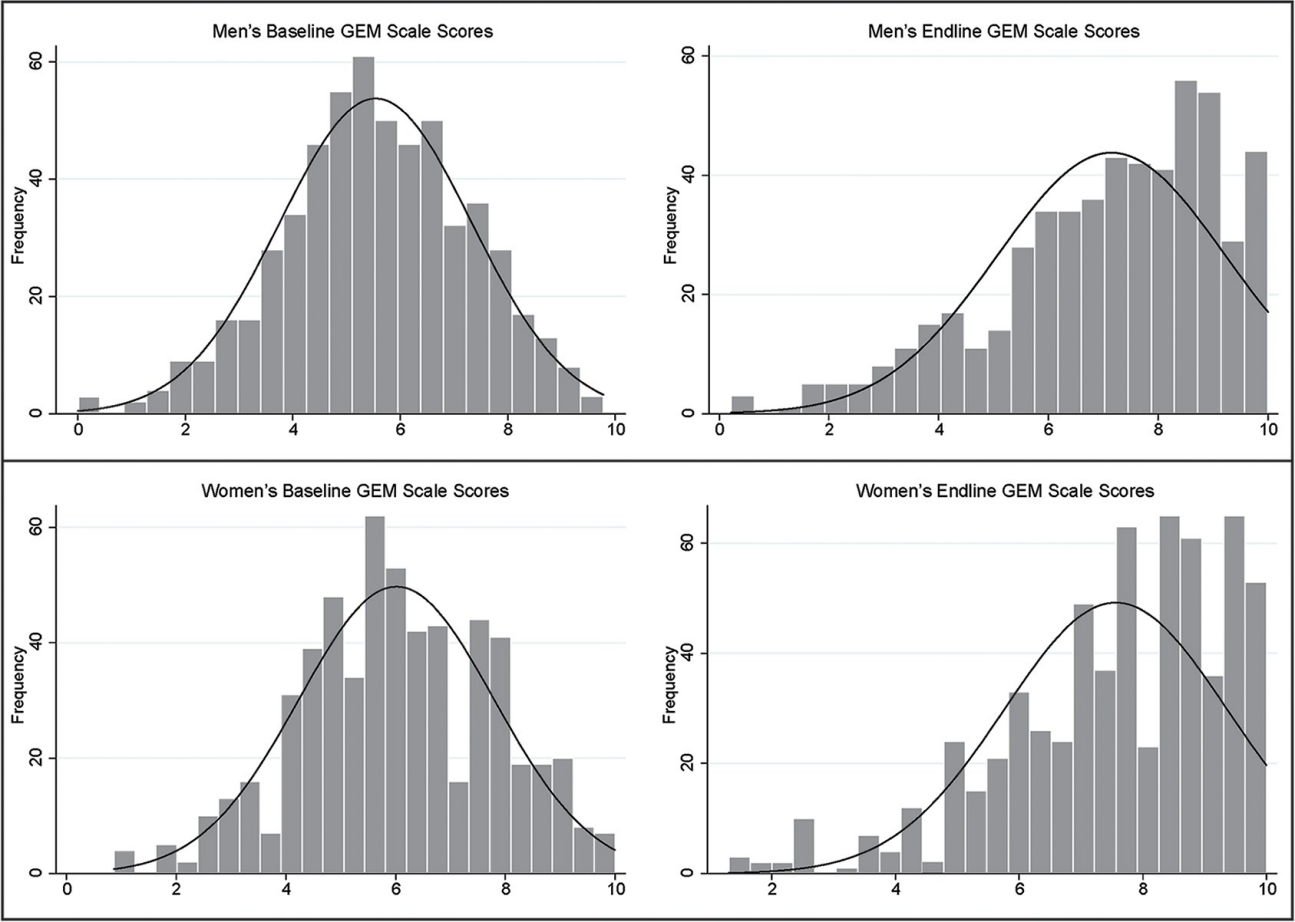
Histogram depicting shifts in the distribution of gender norms scores (GEM Scale) between baseline and endline (irrespective of study arm), among men and women.

Fig 1 depicts the change over time in gender norms, among both intervention and control communities. Fig 2 shows the shift in the distribution of GEM Scale scores over time towards greater equity.

During qualitative research aiming to elucidate quantitative findings, we explored what may have been driving this underlying shift/secular trend in gender norms in both intervention and control communities between 2014 and 2018. First, we explored whether there may have been “contamination” between intervention and control communities. Mobilizers and community members reported only limited direct intervention participation by control community members. And, while they described frequent informal communication between communities included in the trial (to visit social venues and to spend time with friends and family), and some sharing of intervention messages, the messages described related mainly to HIV testing and treatment rather than gender norms or intimate relationships.

We also explored whether there may have been an uptick in media coverage of gender-based violence since 2014. While a number of community members and mobilizers described substantial media focus on this topic in the last few years, including certain highly prominent cases, few thought that there was a notable increase compared with 4–5 years ago (i.e., before the baseline survey), which would be required to explain the shift in gender norms from baseline to endline. As one male community member put it, “*Ja such cases were being reported in the past and even now people are still reporting it*.*”*

Finally, we explored whether there has been any increase in access to media (TV channels; internet) in the study area recently. Participants overwhelmingly confirmed that there has been a recent large-scale increase in access to media, largely via satellite TV channels (which provide access to many previously inaccessible channels) and smartphones. And nearly all, when asked, said that the increase had begun in earnest within the last three to four years.

*There is a change…it was rare to find DSTV [satellite TV service] in our community some years ago… But nowadays every household has DSTV*, *everyone owns smart phones*. *[Since] 2015 or 2014*, *that’s when I started to see a change in our community*. (Male community member)

Most participants said that people buy the satellite TV service, which is seen as affordable: *“Even if you pay R100 [per month] (approx*. *$8 US)*, *there are many channels on that DSTV and more people are using that one*.*”* (Male community member) However, over half of respondents also said that the government had given satellite dishes and/or purchased DSTV for community members with fewer socioeconomic resources: *“government has given people DSTV for free*, *they are busy installing [it] everywhere*.*”* (Male community member) A few participants had received this assistance themselves.

When asked how the increase they had reported in media access may have caused changes in gender norms/relationships between men and women, most participants described how serial dramas (‘soapies’) and talk shows portray couples communicating equitably and resolving conflicts, and mostly depict IPV in a negative light. Most shows mentioned were South African/African-based.

*…in the television there are different stories of love*, *you see two people have different views in their relationship*. *You can see them having arguments then later they sit down to resolve their differences and plead for forgiveness to each other…[this] is able to unite two people who have conflicts…they end up enjoying their relationship again*. (Male community member)

*I always watch those soaps*, *like “Bokithila” and “Nyaninyani”*. *They help people make a decision if there is abuse in their marriage or relationship…There is another soapie…talking about violence or unhealthy relationships…they provide counselling to couples*, *teaching them until they have a solution*… (Female community member)

Of note, we asked specifically about two ‘edutainment’ TV serial dramas that focus directly on gender norms, IPV and HIV: Soul City and MTV’s Shuga Down Under (South African adaptation of Shuga). While most participants had heard of and liked Soul City, they also noted that it has been on for many years and that they themselves didn’t watch it as much in the last few years. None of the participants we interviewed had heard of MTV Shuga.

While the exception, a few respondents described TV negatively impacting views and behavior related to relationships and violence, especially among children. For example, a male community member related: “*I tell my wife when we watch TV that some of the soapies are not good for our children because they will practice what they see on TV*.”

Community mobilizers in both of the FGDs, while agreeing that *“these days people pay a lot of attention to those [TV and talk show] programs”* (FGD-2 participant), also believed that the portrayals critical of IPV and supportive of more gender-equitable relationships were not necessarily translating directly into reduced violence and other risk behaviors. Instead, they explained that Tsima had facilitated personal reflection about the narratives from TV/radio, helping participants connect what they saw to their own lives:

*Mobilizer 1*: *We can see it [referring to the negative effects of IPV] on television but I don’t think we have been touched to such an extent …we just see it from a distance*. *[later segment]* *Mobilizer 2*: *There is a big difference between watching the soapie without someone who can explain much or can tell me the lesson …It is different when I am there facilitating*, *engaging*, *sharing common experiences… coming from the same village*. (FGD-1)

While less common, some respondents also described how increased access to social media via smartphones improved access to and sharing of information. For example, one male community member related how “we learn many things with the smart phones…We get more information through the internet [and we] are using [smartphones] to spread information on social media.”

### Intimate partner violence

IPV results are included in Table 4. About 10% of men reported perpetrating IPV, and about 8% of women reported experiencing IPV, in the last year. IPV prevalence was higher among younger than older respondents. In ancillary analyses, after controlling for socio-demographic characteristics, higher GEM Scale score (i.e., endorsing more equitable gender norms) was associated with lower odds of reporting IPV perpetration among younger and older men (both p<0.01), but was not associated with reported IPV experience among younger or older women.

**Table 4:** Intimate Partner Violence (IPV) at baseline and endline, stratified by study arm, and adjusted effect estimates.

	Weighted %s (95% CI)							
	BASELINE (2014)		ENDLINE (2018)		Unadjusted OR		Adjusted OR^a^	
	N = 869		N = 1,126					
**MEN–IPV perpetration** (last 12 months)	**Control**	**Interv.**	**Control**	**Interv.**	Effect of time	Effect of time x Intervention	Effect of time	Effect of time x Intervention
	n = 190	n = 220	n = 246	n = 259				
**Overall**	12.26%	8.70%	5.53%	6.51%	0.42*	1.74	0.40*	1.85
	(7.50, 19.42)	(7.02, 10.73)	(3.48, 8.72)	(3.35, 12.29)	(0.22, 0.81)	(0.61, 5.00)	(0.20, 0.82)	(0.69, 4.93)
	n = 109	n = 132	n = 151	n = 162				
**Younger** (ages 18–29 years)	16.69%	10.20%	4.24%	5.84%	0.22***	2.47	0.21***	2.61
	(9.03, 28.80)	(7.48, 13.77)	(2.57, 6.92)	(2.95, 11.20)	(0.12, 0.42)	(0.89, 6.88)	(0.10, 0.44)	(0.98, 6.97)
	n = 81	n = 88	n = 95	n = 97				
**Older** (ages 30–49 years)	6.25%	6.16%	7.21%	7.40%	1.17	1.05	1.15	0.96
	(2.35, 15.61)	(4.52, 8.34)	(3.42, 14.58)	(2.8, 18.09)	(0.20, 6.71)	(0.13, 8.35)	(0.21, 6.34)	(0.12, 7.89)
**WOMEN–IPV experience** (last 12 months)	**Control**	**Interv.**	**Control**	**Interv.**	Effect of time	Effect of time x Intervention	Effect of time	Effect of time x Intervention
	n = 221	n = 238	n = 285	n = 336				
**Overall**	6.49%	8.69%	8.37%	6.39%	1.32	0.55*	1.57**	0.53*
	(5.02, 8.36)	(5.31, 13.91)	(5.98, 11.60)	(4.55, 8.90)	(0.96, 1.80)	(0.31, 0.96)	(1.17, 2.10)	(0.31, 0.89)
	n = 102	n = 113	n = 110	n = 146				
**Younger** (ages 18–29 years)	9.64%	12.49%	10.81%	7.98%	1.14	0.54	1.24	0.48*
	(5.77, 15.69)	(8.99, 17.09)	(6.73, 16.91)	(3.78, 16.06)	(0.71, 1.82)	(0.27, 1.08)	(0.75, 2.04)	(0.23, 0.98)
	n = 119	n = 125	n = 175	n = 190				
**Older** (ages 30–49 years)	4.24%	5.21%	7.07%	5.36%	1.72	0.60	2.18*	0.53
	(2.11, 8.35)	(1.59, 15.75)	(4.71, 10.49)	(2.83, 9.91)	(0.82, 3.61)	(0.11, 3.21)	(1.03, 4.62)	(0.09, 3.21)

* p<0.05

** p<0.01

*** p<0.001 Values in parentheses are 95% confidence intervals.

IPV was assessed only among individuals who had a partner; sample sizes are noted in the table.

^a^Controlling for age, education, marital status and received any income in the last three months, and adjusting for the survey sampling design.

“Effect of time” indicates change over time irrespective of the intervention. “Effect of time x Intervention” is the intervention effect—the difference in change over time in intervention vs. control communities.

Regression results for IPV differed for younger (18–29 years) vs. older (30–49) age groups, therefore we describe those results here instead of results for ages 18–49 as a whole. Among younger men, reported IPV perpetration decreased over time (i.e., between baseline and endline) (aOR 0.21; 95% CI: 0.10, 0.44; p<0.001), in both intervention and control communities. Among older men, there was no association between IPV with time or the intervention.

Among younger women, there was a significant intervention effect (i.e., change over time in intervention vs. control) on reductions in reported experiences of IPV (aOR 0.48; 95% CI: 0.23, 0.98; p<0.05). (In unweighted analyses, this aOR was similar at 0.50, but was not statistically significant (95% CI: 0.22, 1.14)). For older women, reported experience of IPV significantly increased over time, irrespective of the intervention (aOR 2.18; 95% CI: 1.03, 4.62; p<0.05). (In unweighted analyses, there was a significant intervention effect on reductions in reported experiences of IPV (aOR 0.29; 95% CI: 0.09, 0.93; p<0.05)).

Qualitative findings around IPV behavior spoke to the positive effects of the intervention on reducing IPV in relationships, reflecting the survey results demonstrating reduced violence for younger women due to the intervention. For older women, on the other hand, the survey showed a general community-based trend as opposed to a link to the intervention; however, this distinction between younger and older women did not emerge in the qualitative interviews.

Tsima participants and intervention staff consistently described how Tsima led to a reduction in IPV largely by promoting critical reflection around violence against women and building skills around healthier couple communication and conflict resolution. As one man explained:

*According to what I have learned in Tsima…we must always communicate with each other*. *I was not communicating with her… if I wanted to do something I was [just] doing it*. *She was always complaining*, *arguing and sometimes I was abusing her physically*. *But Tsima has changed that*, *we are always communicate nowadays*. (Male community member)

Similarly, a mobilizer related the following story about a couple’s eagerness to learn to communicate better and avoid conflict, and the effects a Tsima workshop had in this regard:

*There is someone I know…he was always shouting at [his wife] and abusing her physically*. *The husband attended a [Tsima] workshop and he was asking many questions*… *The following day of the workshop he came with his wife… [Now] there is a big change*, *they are always happy because of Tsima*. (Female mobilizer)

## Discussion

Findings from this study, implemented in the context of a community RCT, demonstrate that gender norms can shift substantially at the community level, in a relatively short period of time, and that such shifts may create an environment in which interventions can find new traction for violence-related behavior change. Across the study area, in both intervention and control communities, there were significant increases in endorsement of equitable gender norms–norms that were quite inequitable at baseline. That these recent broad shifts in gender norms occurred in the study site and can happen in South Africa is further supported by a separate longitudinal observational study by members of our study team (AG, JP) with men ages 20–40 in KwaZulu-Natal province that found a 10% increase in equitable gender norms (p<0.001) between two cross-sectional surveys in 2017 and 2018, which did not vary by participation in HIV prevention interventions [38]. The present study also showed certain changes in IPV, although these findings were less conclusive than those related to shifts in gender norms over time. Younger men’s reports of IPV perpetration decreased over time in both intervention and control communities, while reports of IPV experience by their female counterparts only declined in intervention communities. Taken together, these results suggest progress on the path towards changing gender-related social norms and violence–changes that are critical to reaching Goal 5 of the United Nations 2030 Agenda for Sustainable Development, to achieve gender equality and empower all women and girls [39–41].

The most plausible reason that emerged for the broad shifts in gender norms (and the lack of evidence that these shifts were due to intervention activities), was rapidly-increasing access to TV programming (via satellite dish) and smartphones across the study area, starting since the 2014 baseline survey. Nearly all qualitative study participants described such increased access, which we later confirmed in local and national statistics. For instance, data from the annual Agincourt HDSS census in the study area show that the proportion of households with satellite dishes more than doubled between 2014 to 2018, from 19.6% to 44.4%, after having only increased incrementally since 2001 [42]. And, between 2017 to 2018 alone, smartphone ownership increased from 41.3% to 57.9% (2017 being the first year this was tracked in the HDSS) [42]. National data from the 2016 Demographic Health Survey (DHS) in South Africa showed that three-quarters of adults are watching TV at least once a week, and of the half who use the internet, most do so on a daily basis [43]. Participants also described reasons why exposure to TV/media might lead to changes in views about relationships between men and women, consistently relating how this was exposing people, on a daily/routine basis, to serial dramas and talk shows that negatively portray IPV and model South African couples communicating more equitably and resolving conflicts. While certainly not all effects of increasing access to the TV programming and internet are positive, it seems plausible that current media portrayals of dynamics within relationships between men and women are more equitable and less violent than viewers were previously exposed to in their families and communities. This notion aligns with theoretical work around the social contexts in which transformational interventions occur [44,45]. In particular, Campbell and Cornish (2012) describe how changes in what they call the ‘symbolic context’ can provide easily-accessible representations of ideologies and worldviews–including via the media–that people can draw on in their own lives [44]. It is also consistent with earlier findings from South Africa that national HIV prevention media campaigns, including Soul City, were associated with positive shifts in social norms and health-seeking behavior [46].

Turning to findings about changes in IPV, survey findings suggested that the intervention led to reductions in reported IPV experience among younger women. While qualitative results largely supported this finding, it should also be interpreted with caution particularly given non-significance of the effect estimate in the unweighted analysis (although similar in magnitude). Younger men in both intervention and control communities reported reductions in IPV perpetration, leading to a null intervention effect. This mirrors findings from a community RCT of Sonke Change, an intervention similar to One Man Can (from which Tsima was adapted) implemented in Diepsloot (a township near Johannesburg) around the same time as Tsima (2016–2018) [21]. This trial found substantial reductions in men’s reported IPV perpetration over the two years, in both intervention and control communities; for example physical IPV perpetration declined from about 40% to 25% in both trial arms (women were not surveyed) [21]. It could be that the increasing media exposure described above directly impacts younger men’s IPV behavior even without an intervention present. Alternatively, perhaps younger men underreported IPV perpetration because of new community norms and/or media messaging contrary to such behavior; this would align with our finding that younger women in control communities did not report a decline in IPV. The lack of change in IPV among older men due to either time or the intervention, and the increase over time among older women (albeit small in magnitude), also require further examination. It may be that there was less room for decline among older respondents, among whom only about 4–6% reported IPV perpetration/experience at baseline, vs. 10–17% among younger respondents.

Qualitative findings suggested an intervention impact on reducing IPV, among both women and men (differential impact by age group was not explored in detail). Two main reasons why Tsima may have prompted such reductions emerged from the interviews: 1) critical reflection about gender equity and IPV, and 2) skill-building to improve communication and reduce conflict in their own relationships. As noted previously, Tsima explicitly included activities to promote such critical reflection and skill-building, particularly in the 2-day workshops, which men and women attended together [15,47]. Respondents also described how messaging/modeling on TV, while a consistent presence in people’s lives, remained ‘distant’–and how facilitation and direct engagement in Tsima helped men and women translate these messages into new patterns of interaction in their relationships.

These findings echo previous experience from gender-transformative interventions that while community mobilization-only and mass media interventions can produce changes in individual and collective attitudes, critical reflection and skill-building around gender equity and violence prevention, usually imparted in smaller and more intensive groups, is often required to support notable change in related behaviors [9,11,13,19,48,49]. For instance, Viitanen & Colvin (2015), in a synthesis of qualitative findings from five gender-transformative interventions in South Africa, found that a key to men’s receptivity was seeing the messages in the context of their own lives, and the rich dialogues that resulted [50]. Still, a rigorous evaluation of the One Man Can program, from which Tsima was adapted, implemented in 2012–14 in the same study area as the present study (but in different intervention communities), did not find effects on IPV behaviors despite demonstrating an effect on positively changing gender norms [7]. Improved impact on IPV in Tsima vs. One Man Can could owe to Tsima’s more explicit tailoring of content to engage both men and women, more intensive focus on negotiating HIV-related disclosure and service uptake, or its longer duration (three vs. two years). It could also be that population-level shifts in gender norms since 2014 provided new traction for Tsima to reduce IPV behaviors among some individuals.

There are several important implications of our findings. The first is the need for greater attention to the (often rapidly changing) social contexts in which interventions are carried out–including the “digital transformation” currently underway across the African continent and widely seen as critical to development [51,52]. Indeed, increasing equitable access to information and communication technologies features prominently in the UN Sustainable Development Agenda, including under Goal 5 to achieve gender equality [39]. Yet in the discourse around gender-transformative approaches in public health, the effects of this digital transformation, including how media shapes gendered social expectations, is much less prominent. Certainly, many health-related social and behavior change interventions have employed mass media and ‘edutainment’ approaches, and others have sought to use mobile phones/SMS to support social and behavior change–often with success. However, there remains an inattention to the implications of broader social transformations underway, perhaps nowhere faster than in Africa, and how these could be harnessed by public health initiatives to promote healthier behaviors.

A second key implication is the need to scale up facilitated discussion that fosters critical reflection around gender, power, and IPV, as well as skill-building around shared decision-making and conflict resolution in relationships. Both women and men in our study described their eagerness for this guidance, and willingness to engage constructively, often together as a couple, to achieve more equitable and violence-free relationships. Several evidence-based group workshop curricula are available to do this [4,5,7,10,11,53], including those employed in the present intervention; these would need to be scaled up and monitored closely for fidelity. It may also be possible for a brief activity (generic, or tailored based on a relationship dynamics screening) to be incorporated into other platforms that already reach individuals and/or couples, such as in healthcare settings, online chat groups, and workplace programming.

This study has several limitations. While the cross-sectional nature of the surveys has benefits in terms of gauging community-level change, not assessing the same participants over time makes it more difficult to link changes to the intervention. And, at endline there were certain differences by timepoint or intervention status. While these were controlled for in our analyses, there may be other unmeasured differences. Data are based on self-report, which could lead to social desirability bias in responses. In particular, the discrepancy in control communities between men’s decreased reports of perpetrating IPV and women’s increased reports of experiencing IPV, may be due to social desirability bias in which men underreported IPV perpetration. Finally, due to the number of tests of associations carried out in this study, there is a potential for increased detection of significant associations when in fact they were due to chance.

## Conclusion

Societal-level gender norms have long been seen by policy makers as intractable, as well as unquantifiable–leading to a lack of due attention and funding commitments [40]. Our results help counter this narrative: we found broad, large-scale shifts towards endorsement of more equitable gender norms, which also suggests the ability of available measures to capture this change. Such shifts in normative environments could provide new traction for HIV and IPV prevention, if coupled with related critical reflection and skill-building. This is a rich area for future research that could guide the way towards translating shifts in the social context into lasting positive effects in men’s and women’s lives.
